# Progress in Neurology

**Published:** 1903-08-15

**Authors:** 


### Progress in Neurology.

(*Concluded from page* 332.)

**Meningitis.**—Wall[3] has recently analysed the records of 22 cases of meningitis due to the diplococcus intracellularis of Weichselbaum (as proved by bacteriological methods) to determine the symptomatology. There is no narrow age limit—the ages varied from two months to 35 years, and there is no evidence of the actual mode of infection or of spread by contagion. The course of the disease is marked by two stages—(1) that of acute meningitis, and (2) that of internal hydrocephalus. The characteristics of the first stage are sudden or rapid onset in the majority of cases, the most common initial symptom being headache or convulsions followed by a rapidly developing mental disturbance. The temperature is high, the pulse frequent and regular, vomiting is fairly frequent at the onset, but does not recur until the second stage is reached. Wasting is early. Rigidity of the neck with resistance to forward flexion of the neck, but not to rotation, is practically constant. Spinal opisthotonos is not common. Kernig's sign is present in the majority of cases. Optic neuritis is uncommon. The duration of the first stage in the fatal cases varies from three to 12 days. Those patients who recover either pass into the second stage of hydrocephalus, the symptoms of which develop during the third week, or pass into a condition of somewhat tedious convalescence. The second stage is characterised by vomiting, occasional retraction of the upper lids, irregular rises of temperature from a normal base line, mental disturbance apparently associated with the rise of intra-cerebral pressure. Lumbar puncture at this stage shows the cerebro-spinal fluid to be under considerable tension, and relief of the tension is often followed by an amelioration of the symptoms; as a method of treatment this is of considerable value. Post-mortem examination of cases dying in the first stage shows purulent meningitis without distension of the cerebral ventricles; those dying in the second stage show distension of the ventricles with fluid, with collections of lymph in the basal lymph cistern. The purulent exudate lies between the pia mater and the arachnoid, and in the first stage it is general in its distribution. The rigidity of the neck is probably to be explained as protective and preventing the mechanical disturbance of the inflamed arachnoid. In antero-posterior flexion of the head the cerebellum slides on the medulla, and rigidity of the neck prevents such movement. The production of hydrocephalus in the second stage is of doubtful origin; the balance of evidence seeming to point against the truth of any mechanical theory, whilst the most probable theory is that which, recognising the arachnoid as a secreting membrane, supposes that there is some disturbance in the balance between secretion and absorption just as in the case of pleural effusions. "Posterior basic meningitis," "cervical opisthotonos of infants," and "epidemic cerebro-spinal meningitis" are probably identical, and should be included under the general term "acute general leptomeningitis."

**Trigeminal Neuralgia.**—Six Gasserian ganglia which had been removed from cases of severe and intractable neuralgia have been examined by Schwab.[4] The specimens were fixed and hardened while fresh and examined by the latest methods. In some of the ganglia neuritic changes were observed in the peripheral branches, but only in those cases in which a peripheral operation had previously been performed. The nerve cells in the peripheral parts of the ganglion showed changes varying from a slight degree of chromatolysis to profound changes of total cell disintegration and nuclear migration. In three of the ganglia pigmentation was observed round the nuclei of the nerve cells quite different in character and distribution from that present in normal conditions. In one of the ganglia a sclerosis of the tissue with excess of fibrous formation was found. The disappearance of the symptoms of neuralgia when the ganglion is removed or when the root connecting it with the brain is divided shows that the ganglion is the seat of the disease. The nerve cells exhibit signs and changes of prolonged irritation and over-stimulation, in some respects closely resembling the appearances produced (artificially) by electric stimulation for several hours. The agent in cases of disease is probably of some toxic nature circulating in the blood and having a selective action on the nerve cells of the Gasserian ganglion.

[3] Lancet, May 2, 1903. [4] Jour. of Nerv. and Mental Disease, Feb. 1903.

